# Effectiveness of haptic feedback devices in preclinical training of dental students—a systematic review

**DOI:** 10.1186/s12903-023-03410-3

**Published:** 2023-10-10

**Authors:** Shankargouda Patil, Shilpa Bhandi, Kamran H. Awan, Frank W. Licari, Marco Di Blasio, Vincenzo Ronsivalle, Marco Cicciù, Giuseppe Minervini

**Affiliations:** 1 College of Dental Medicine, Roseman University of Health Sciences, South Jordan, Utah 84095 USA; 2 Department of Medicine and Surgery, University Center of Dentistry, University of Parma, 43126 Parma, Italy; 3 Department of Biomedical and Surgical and Biomedical Sciences, Catania University, 95123 Catania, CT Italy; 4 Multidisciplinary Department of Medical-Surgical and Dental Specialties, University of Campania Luigi Vanvitelli, Naples, Italy; 5 Saveetha Dental College & Hospitals Saveetha Institute of Medical & Technical Sciences, Saveetha University, Chennai, India

**Keywords:** Haptics, Preclinical operative dentistry, Psychomotor skills, Simulation, Virtual reality

## Abstract

**Background:**

Acquisition of psychomotor skills is of utmost importance for competent preclinical restorative dentistry. Recent advancements in haptic feedback technology have been incorporated into preclinical dental education to augment the conventional phantom head-based training.

**Objective:**

This systematic review aims to assess the effectiveness of haptic feedback device, Simodont, in improving the skill development and learning outcomes of dental students during their preclinical training.

**Materials and methods:**

Electronic databases Web of Science, Scopus, PubMed were searched for relevant studies since inception up until March, 2023. Only English language studies that assessed the effectiveness of haptic feedback devices in preclinical dental education were included. We excluded studies that did not use Simodont as the haptic feedback device or did not involve preclinical restorative work. Study quality was assessed using the revised Cochrane risk of bias tool and ROBINS-I. The primary goal of the study is to evaluate the efficacy of Simodont as a complementary training modality for dentistry students.

**Results:**

Results from 9 high-quality studies were analyzed and synthesized to evaluate the overall impact of haptic feedback devices on various aspects of preclinical training. The studies were conducted on 826 undergraduate dental students enrolled in various years of their training across dental colleges and universities in different parts of the world. A majority of studies showed some concerns regarding risk of bias. Haptic feedback devices added a new layer to Virtual Reality (VR) through the perception of touch and force feedback. It assisted junior dental students improve their psychomotor skills and movement skills. Instantaneous feedback on the students' performance helped enhance their self-assessment and correction, and also eliminated the subjectivity of evaluation. Data derived from virtual simulators helped stratify dental students and predict their clinical performance, providing an opportunity to tailor the learning process to meet individual diversity in students' expertise.

**Conclusion:**

Based on the limited evidence available, Simodont was effective in preclinical training of dental students, offering advantages such as unlimited reproducibility, objective evaluation of preparation by computer assessment, and cost reduction. And further studies are warranted to explore the incorporation of patient's oral environment simulation for better skill training.

## Introduction

Dentistry requires the development of precise manual dexterity. In contrast to many other health sciences disciplines, dentistry primarily focuses on practical applications, including the manual administration of restorative treatments. The primary focus and central competence of preclinical operative dentistry is the development of psychomotor abilities among dental students. This aspect receives the majority of curriculum time during the preclinical phase [1].

Students are often instructed on phantom heads in conventional preclinical training. Phantom head or mannequin training is a pedagogical strategy used in dental education that use a simulated human head or mannequin to give students with a safe and genuine environment in which to improve their procedural abilities. These training models are designed to mimic the physical characteristics and anatomy of a human head, including the teeth, gums, and jaw, allowing students to practice procedures such as tooth extraction, filling, and crown preparation. It allows students to practice their motor skills, develop their dexterity and hand–eye coordination, and improve their understanding of dental anatomy and oral physiology [2]. However, it lack the realistic tactile sensations experienced during actual clinical procedures. Also, plastic teeth do not sufficiently replicate the variability of natural teeth and the significant accumulation of plastic waste associated with their use presents a substantial environmental concern. It is increasingly difficult to obtain natural human teeth due to ethical constraints [3–5]. In conventional training, the assessment process often relies on the retrospective evaluation of a student’s skill and practises after their training sessions. This disparity between preclinical simulation and real-world practice can lead to challenges for students when they transition to treating real patients [6–8].

The integration of haptic feedback technology in dental education aligns with the evolving educational landscape, emphasizing evidence-based approaches and technology-driven pedagogy. Haptic feedback devices have been proposed as a complementary modality for conventional preclinical training methods, such as working with mannequins or extracted teeth [9]. Haptic technology, also referred to as kinaesthetic communication, is capable of eliciting a proprioceptive response through the use of vibration and force in coordination with physiological motions. This form of training provides dental students with the opportunity to practise procedures on virtual patients using haptic simulators that provide realistic tactile force feedback. The Force Feedback is a cutting-edge technology that revolutionizes dental training. It employs haptic feedback to provide realistic tactile sensations during virtual dental procedures. When a student interacts with the virtual tooth using a dental instrument, the haptic device generates force feedback that simulates the resistance and pressure experienced in real-life clinical settings. This dynamic feedback allows students to develop and refine their motor skills, hand–eye coordination, and dexterity while performing various dental tasks [10].

Haptics dental suites displays the tooth and the instrument in three-dimensions in true size on screen. The tooth is presented digitally as a series of voxels of varying density [11]. Each point on the tooth reacts differently to the contact of a drill, with force being transmitted through the haptic device to the user. The force transmitted is proportional to density values of each voxel that makes up the three-dimensional tooth [12]. The user can receive different force feedback depending on the tooth and angle of the tooth, resembling a real-life clinical situation. Haptic technology is used with many phantom heads and mannequins to simulate working with real dental instruments and materials, giving students a more realistic and effective training experience [13]. The ability to practise operations indefinitely in a safe and controlled environment without the risk of harming a living patient is a major advantage of haptic-based training [14]. The use of haptic technology in dental education has been shown to improve student’s motor skills and increase their confidence in performing procedures in a clinical setting [10, 15].

Simodont is a dental training simulator that utilizes haptic technology to provide realistic tactile feedback while performing virtual dental procedures [16]. Simodont training system is widely used in dental schools and training centers around the world. The system can be customized to simulate various dental procedures and conditions, providing students with a comprehensive and immersive educational experience [17]. The system utilizes advanced sensors and algorithms to track and analyze a variety of parameters such as the pressure and angle of the instrument, the speed and trajectory of movement, and the duration of the procedure [18]. The system can supervise a student’s preclinical work, identifying if a student is working on the wrong tooth or inadvertently inflicting damage to the virtual gums or other soft tissues. This data is used to provide personalised feedback to the student, such as identifying areas where they may be applying too much or too little pressure, or where they may be deviating from the proper trajectory. This feedback can be customized to the individual student's needs and skill level, providing a more efficient and effective learning experience [19].

Few published studies have systematically examined the effectiveness of preclinical haptic dental training of undergraduate students [20, 21]. Existing knowledge regarding virtual training primarily focuses on the assessment of augmented reality or virtual reality systems, with limited systematic reviews delving into a comprehensive analysis of a singular haptic dental device specifically designed for preclinical training of dental students. The objective of this study was to investigate the impact of haptic feedback devices on the development and acquisition of psychomotor skills in dental students during preclinical training by analysing current literature, which included both experimental and observational studies.

## Materials and methods

### Search strategy

This systematic review was conducted in accordance with the guidelines outlined in the Preferred Reporting for Systematic Reviews and Meta-analysis (PRISMA) statement [22].

The focused question was “*Are Simodont haptic feedback devices effective in the clinical training of dental students?”.*

Inclusion criteria consists of undergraduate dental students from all over the world who are in different stages of their training and come from different dental colleges and universities. In our study, the intervention is the use of haptic feedback devices, like the Simodont haptic training system, for preclinical training of dental students. The comparison group is made up of students who do their preclinical training without using haptic feedback devices. The primary outcome in the study is the development and acquisition of Psychomotor skills/clinical skills/dexterity in dental students during preclinical training. And we considered numerous types of studies, like randomised control studies, controlled clinical trials, and cohort studies.

The criteria for inclusion are written in the PICOS format, which stands for Population, Intervention, Comparison, Outcome, and Study type. This framework is used to precisely articulate the study selection criteria and research question in systematic reviews and meta-analyses.

### Inclusion criteria

(P) Population: Dental students.

(I) Intervention: Training using Simodont haptic feedback devices.

(C) Control: Phantom head or no control.

(O) Outcome: Psychomotor skills/clinical skills/dexterity.

(S) Study type: Randomised control studies, controlled clinical trials, cohort studies.

### Exclusion criteria

Case reports, conference proceedings, systematic reviews, opinion articles, letters to the editor, case reports and articles in languages other than English were excluded.

### Search strategy and study selection

A comprehensive search was conducted on electronic databases including Pubmed, Scopus, and Web of Science. The search query for PubMed included a combination of Medical Subject Headings (MeSH) terms and relevant keywords related to haptic interfaces and technology, dental students, and Simodont. The search strategy for Scopus included a combination of relevant terms related to haptic technology, dental training, and Simodont; whereas for Web of Science, the search query combined the keywords "haptic" and "dental students". The search was limited to papers published in the English language, with no constraints imposed on the start date. The search was performed in March 2023. After executing the search queries, a total of 64 articles were retrieved from PubMed, 3 articles from Scopus, and 17 articles from Web of Science. Details of the search strategy are provided in Table 1. The first screening of search results for study selection was carried out by two independent reviewers (SP and MDB), who removed duplicates and non-relevant publications. Subsequently, titles and abstracts of studies were screened to determine their eligibility, and discrepancies were resolved through consensus with a third author. Subsequently, full-text review of the shortlisted studies was conducted based on pre-defined inclusion criteria, and a third author (XZ) was consulted for the final decision in case of any contention. Furthermore, manual supplementary searches were conducted on references of the selected articles in order to identify any additional eligible studies. The details of the selected studies are provided in Table 2.

**Table 1:** Search strategy

Database	Search query	Results
PubMed	("haptic interfaces"[MeSH Terms] OR ("haptic"[All Fields] AND "interfaces"[All Fields]) OR "haptic interfaces"[All Fields] OR "haptic"[All Fields] OR "haptic technology"[MeSH Terms] OR ("haptic"[All Fields] AND "technology"[All Fields]) OR "haptic technology"[All Fields] OR "haptics"[All Fields] OR "haptical"[All Fields] OR "haptically"[All Fields]) AND ("students, dental"[MeSH Terms] OR ("students"[All Fields] AND "dental"[All Fields]) OR "dental students"[All Fields] OR ("dental"[All Fields] AND "student"[All Fields]) OR "dental student"[All Fields])	64
	"simodont"[All Fields] AND ("students, dental"[MeSH Terms] OR ("students"[All Fields] AND "dental"[All Fields]) OR "dental students"[All Fields] OR ("dental"[All Fields] AND "student"[All Fields]) OR "dental student"[All Fields])	19
Scopus	TITLE-ABS-KEY ( Haptic AND dental AND training)	3
Web of Science	Haptic (All Fields) and dental students (All Fields)	17

**Table 2:** Characteristics of the selected studies

Author	Sample Size	Level	Study design	Pretraining	Version of Devices	Intervention	Evaluation items	Assessment	Results	Inference
de Boer et al*.* (2017) [23]	62 dental students	1st year	Grp 1: students who practiced without effect of force feedback Grp 2: students who practiced with effect of force feedback	Practice sessions—the cross preparation with a dental teacher available for questions	Moog Simodont dental trainer (Nieuw-Vennep, the Netherlands)	Students practiced with or without force feedback depending on the group they were assigned to, and then completed an assessment task involving cross preparation. All students were tested under both conditions, with half of the group starting with force feedback and half without, assigned randomly	To pass test 1 or test 2, three of five (ie, cross) preparations had to be successfully completed within 45 min. The maximum number of permitted manual dexterity preparations during the tests was 5	Data collected during both tests were statistically analyzed	Students failed test without Force Feedback, confirming its necessity for high precision manual dexterity tasks in dentistry. Only FFB users passed the tests	The results suggest that effect of force feedback is important for performance in a virtual learning environment and essential for satisfaction. FFB is required to perform on the high precision level that manual dexterity tasks in dentistry require
Mirghani et al*.* (2018) [24]	289 Dental Students	Year 1 (n = 92), Year 3 (n = 79) Year 4 (n = 57) Year 5 (n = 61)	All participants engaged in 6 manual dexterity exercises	Students received an instruction sheet and verbal guidance from a tutor on system operation, including how to turn it on, log in, and select tasks. They adjusted the chair and unit to their comfort and wore stereoscopic spectacles. Questions were encouraged throughout the training	Simodont ‘courseware’ software (developed by the Academic Centre for Dentistry Amsterdam (ACTA), Amsterdam, Netherlands)	Geometric Shapes- The task involved the use of a dental hand piece to remove a target ‘red zone’, presented as a cross-shape in the middle of a block, whilst attempting to minimise removal of leeway zones (the ‘safe’ outer areas of the block) as much as possible	Real-time feedback on performance was recorded- a percentage score for each of the following: Target (task completion percentage) Error scores (Leeway Bottom, Leeway Sides, container bottom and container sides) Drill Time (in seconds)	Measured performance on four outcome variables: Time (in seconds), Leeway Bottom, Leeway Sides (quantified as percentages) and finally, a Composite Score that captured speed-accuracy trade-offs in performance	Simodont has shown sensitivity to performance differences between novice and experienced students Statistically significant differences were found between novice (Year 1) and experienced dental trainees (operationalised as 3 or more years of training), but no differences between performance of experienced trainees with varying levels of experience	The study provides evidence that the Simodont is a valid tool for assessing motor skills in dental students and can differentiate between different levels of real-world dental experience. The results showed that the performance of dental students improved as their level of experience increased, and the time taken to complete the task decreased as their level of experience increased
Al- Saud et al*.* (2020) [25]	72 dental students (46F,26 M)	Year 4	The study was conducted with second-year undergraduate dental students, and their performance on a VR haptic simulator was compared to their subsequent clinical performance involving patients two years later		Simodont	crown preparation using Simodont and typodont	simulator to measure the performance of second-year undergraduate dental students on a variety of simulated abstract manual dexterity tasks. The simulator was used as part of a formative assessment, and the students were left to their own devices and could practice as much or as little as they liked	The study compared the performance of the students on the VR haptic simulator to their subsequent clinical performance involving patients two years later	Mean performance on simulated manual dexterity tasks 2 years earlier could explain 14% of the variance in clinical performance scores in year 4 of dental study. This measure was more effective than a traditional typodont test, despite the typodont test being conducted preclinically 1 year prior to the clinical measure	Simodont shows promise in predicting clinical performance and identifying individuals who may need early training support
de Boer et al*.* (2019) [26]	126 dental students	Year 1	Grp 1: Low levels of force feedback and Standard levels of force feedback Grp 2: Standard levels of force feedback and Low levels of force feedback Grp 3: High elevels of force feedback and Standard levels of force feedback Grp4: Standard levels of force feedback and High levels of force feedback	Participants trained for 3 months during their scheduled educational time with standard levels of force feedback on 3 geometric figures to improve fine motor skills	Simodont dental trainer (Moog, Inc, Nieuw-Vennep, the Netherlands)	Students prepared the "block cross-figure" using standard and low or high levels of force feedback, depending on their assigned group	Effect of practicing with standard force feedback on student performance was examined, along with the ability to transfer skills from one force feedback level to another	five attempts—the student had to pass three of them for each level of effect of force feedback	Even inexperienced students can transfer their manual dexterity skills from one level of force feedback to another. This suggests that once the skill is mastered in virtual reality, it can be transferred to the real world. Therefore, it is important to incorporate virtual reality education into dental curricula	The study highlights the importance of accuracy in haptics (sense of touch/feeling) in a simulation environment and the need for further research to investigate the transfer of skills from reality to VR
Murbay et al.(2020) [27]	32 students	Year 2	Grp 1: Received feedback from machine only Grp 2: Instructor verbal feedback only Grp 3: Instructor and device feedback		Moog Simodont dental trainer (VR)	Class I cavity preparation on plastic mandibular molar tooth	•underprepared/overprepared, •centeredness •contour smoothness •depth of preparation •convergence and divergence of walls •line angles •treatment execution	Three assessors visually assessed the preparations under 2.5 × magnification, while also saving the preparations as STL files for digital assessment	In both manual and digital assessment, students who had practiced with the virtual dental trainer showed better scores compared to the control group	The virtual reality simulator may be a valuable adjunct in the UG direct restorations course and for student remedials
Cecilie Osnes et al*.* (2021) [28]	112 dental students. 17 Clinicians	Year 1 students and clinical teaching staff	Group 1: Dental Students Group 2: Clinicians	Users were introduced to the exercise with a prewritten verbal introduction displaying their progress live on screen. The exercise could be restarted or repeated within a 15-min time frame, and the color and live score helped users understand the task	Simodont	Generated caries lesions using unique shapes as "seed images". Two blocks were used—one for introduction containing green caries and one for testing without any discolouration	Participant precision score was calculated using the number of voxels drilled in each of the segments: enamel, dentine, ADJ caries (including any unsupported enamel), (deep) caries and pulp	Perfect score for the precision of tooth preparation was achieved by removing unsupported enamel and ADJ caries, which represented approximately 7% of the complete block. Any additional material removed, except for deep caries, would result in a penalty to the score. The precision score was calculated as the percentage of ADJ caries removed minus the percentage of enamel, dentine, and pulp removed	The exercise may be a useful tool for assessing conceptual understanding of caries removal. Clinicians were significantly more precise than students in removing caries without excessively removing the non-carious parts of the block p = 0.009. Clinicians removed significantly more caries at the ADJ (ADJ caries) than students Fourteen (82%) clinicians and 69 (62%) student participants removed more than half of the deep caries available	Haptic simulation exercises may be a useful tool for assessing understanding of the concept of caries removal
Ignacio Aliaga et al*.* (2020) [29]	82 students	1st year dental students reevaluated in third year	A total of 82 students who completed the first year of dentistry were followed for 2 years. Their performance on the same task (i.e., cavity preparation of three figures in the Simodont and methacrylate blocks) was then reevaluated in the third year	Enrolled students had performed preclinical practice first in MBs and then in the SDT. Students received a brief explanation	Simodont (Moog Inc., Nieuw-Vennep, Netherlands) with software 3.18.4	Students sequentially prepared three cavities with geometric shapes (bar, circle, and cross) in 15 min, removing simulated caries (red) from each figure through the software	1. Percentage drilled segment target 2. Percentage drilled leeway bottom (PDSLB) 3. Percentage drilled leeway sides (PDSLS) 4. Percentage drilled container bottom (PDSCB) 5. percentage drilled container sides (PDSCS), where variables 2 to 5 reflect students’ errors 6. session time, which is real time (seconds)	The same professor evaluated the students performance first in first year and then in third year	The Simodont practice can be reliably evaluated. Preclinical methodologies of Simodont detected improvements in the manual skills of first- and third-year dental students	Both methodologies can detect manual skill improvement in dental students. Additionally, the Simodont practice can be reliably evaluated
Hattori et al*. (2022) * [30]	30 dental students (12 M, 18F)	6th year	Grp1:dental students using the haptic simulator Grp 2: dental students using a conventional mannequin simulator	Students were instructed on how to operate the haptics simulator prior to tooth preparation. Following a 10-min period of explanation, subjects were given free practice time to become familiar with the device	Simodont" (Nissin Dental Products Europe BV, Nieuw- Vennep, Netherlands)	full cast crown of the right first molar in the mandible	Each item was scored on a 5-point rating scale •the occlusal surface •margin design •Surface smoothness •Taper angle •Total cut volume •Overall impression of the products	Three evaluators with more than 10 years of university teaching experience evaluated the products of both haptic and conventional simulators	The scores of the haptics simulator were lower than those of the conventional type for several evaluation items. The conventional simulator showed higher values for Margin design, Surface smoothness, and Total cut volume, and the difference between the simulators was significant (P < 0.05) Margin design and Total cut volume showed a significant difference in the score between haptics and conventional simulators (P < 0.05)	This study suggests that the unique characteristics of virtual reality, such as the simulated cutting sensation and the simulated three-dimensional images created by stereo viewers, affect operators’ performance and evaluators’ perception. Therefore, it is important to develop an educational program that is conscious of the features of each simulator
Farag and Hashem (2021) [31]	21 female dental students	3rd year	Prepared cavities before and after Haptic virtual reality simulation training for each student were used as an assessment tool for the students’ psychomotor skills	Lecture and hands-on demonstration	Simodont	Training on geometric Shapes Testing Class I cavity preparation on plastic mandibular molar tooth	•Occlusal outline •Pulpal floor •Buccal and lingual walls •Internal line and point angles	Two evaluators independently assessed based on design features	statistically significant increase in the overall marks after haptic virtual reality simulation training there was an improvement in all evaluation criteria scores after Haptic virtual reality simulation training	Improved overall performance in psychomotor skills was found after haptic virtual reality simulation training

### Data extraction

The data extraction form used in this systematic review was developed based key attributes and characteristics that were relevant to our research, including study details (e.g., authors, publication year, country of origin), study population (e.g., dental students' stage of training, institution), intervention details (e.g., type of haptic technology used, duration of training), and outcome measures (e.g., assessment of psychomotor skills, clinical performance). Additionally, we included specific criteria for assessing the risk of bias in the included studies. The process of data extraction from the shortlisted studies was conducted by two reviewers (XX and XX) who worked independently. To ensure the correctness of the extracted data, a third author (XX) validated the results. The pertinent attributes of the studies that were included, such as the names of the authors, the year of publication, the country of origin, the methodological details the sample size, the treatment regimen, and the duration, were manually extracted and recorded in a customised template.

### Assessment of study quality

The quality of the selected studies was by two reviewers (SP and FL) individually. They assessed the risk of bias for randomized studies using the revised Cochrane Risk of Bias tools for randomised trials (RoB-2) and non-randomized controlled studies using the Risk of Bias in Non-randomised Studies of interventions (ROBINS-I) tool [32, 33]. Any disagreements between the reviewers were resolved through discussion or by consulting a third reviewer (GM).

RoB-2 was used to assess the risk of bias in studies by assessing five domains: bias arising from the randomization process, bias due to deviations from intended interventions, bias due to missing outcome data, bias in the measurement of the outcome, and bias in the selection of reported results. Each domain was evaluated through a set of signaling questions to identify potential sources of bias in the study. The responses to the signaling questions were used to assign a judgment of low, high, or some concerns regarding the risk of bias for each domain.

In ROBINS-I, signaling questions focusing on seven domains during pre-intervention, at-intervention, and post-intervention, were used to evaluate the studies. The evaluated domains encompass confounding variables, participant selection, intervention classification, deviations from intended interventions, missing data, outcome measurement, and selection of reported results. For each domain, specific criteria are used to evaluate the risk of bias, and the overall risk of bias is rated as low, moderate, serious, or critical.

### Quality of evidence for outcomes in Summary of Findings table

The GRADE evidence grading system, which is described in Sect. 12.2 of the Cochrane Handbook for Systematic Reviews of Interventions, was used to rate the quality of the evidence for each outcome in the Summary of Findings [32]. One of the authors used the GRADE system, and subsequently discussed with the other two authors to reach a consensus on the quality of evidence for each outcome. The criteria used for downgrading the quality of evidence included five domains: risk of bias, inconsistency of results, indirectness of the evidence, imprecision of the results, and publication bias.

## Results

**Fig. 1:**
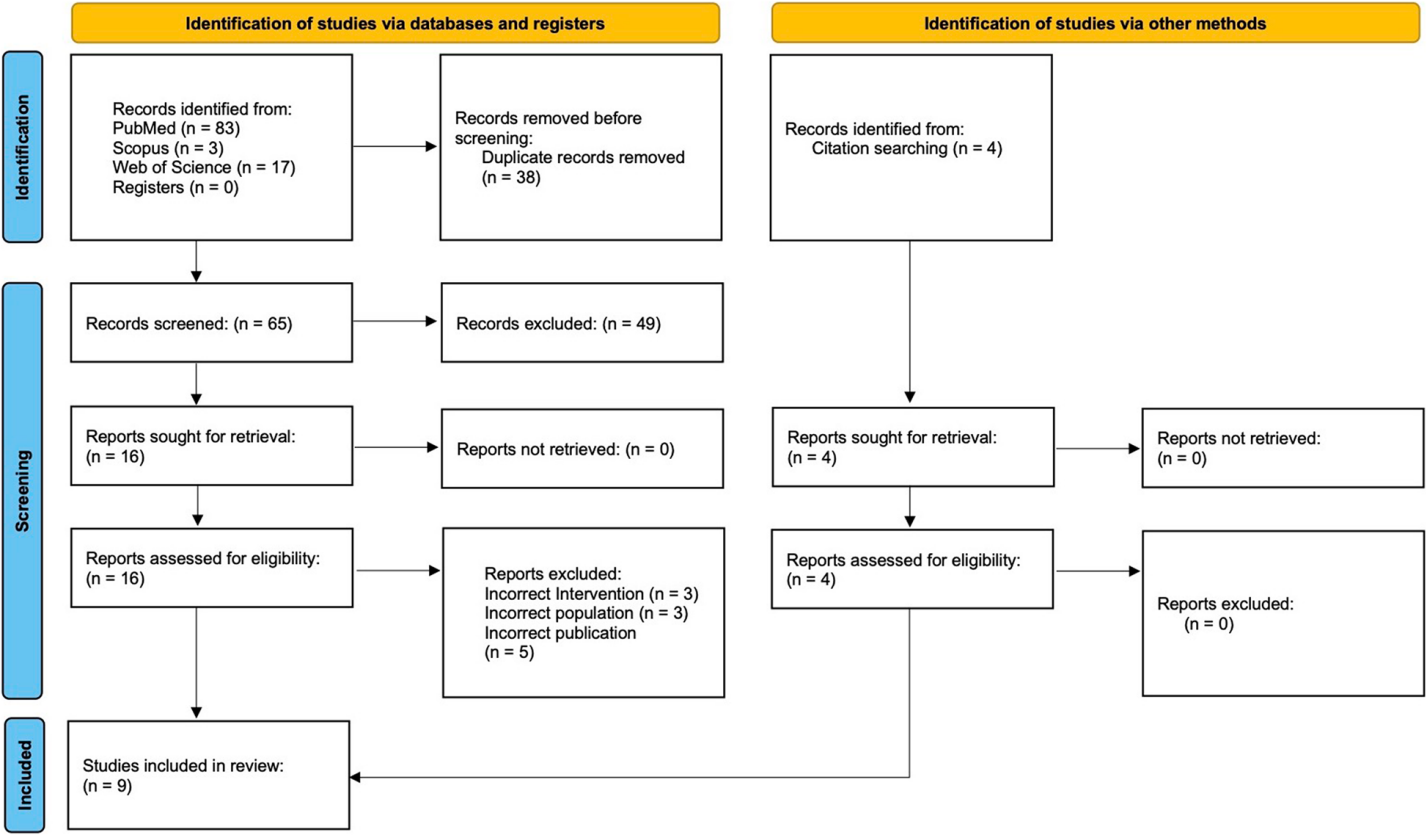
PRISMA flow chart

We found 103 results in our first pass through the database searches. After removing 38 duplicates, the remaining papers were screened on the basis of title and abstract for eligibility. Sixteen full-length papers were obtained for assessment. Citation searching of papers led to an additional four papers. Finally, nine articles published between the years 2017 and 2023 were included in the present review [23–31]. The PRISMA flow diagram is shown in Fig. 1.

### Quality assessment

**Fig. 2:**
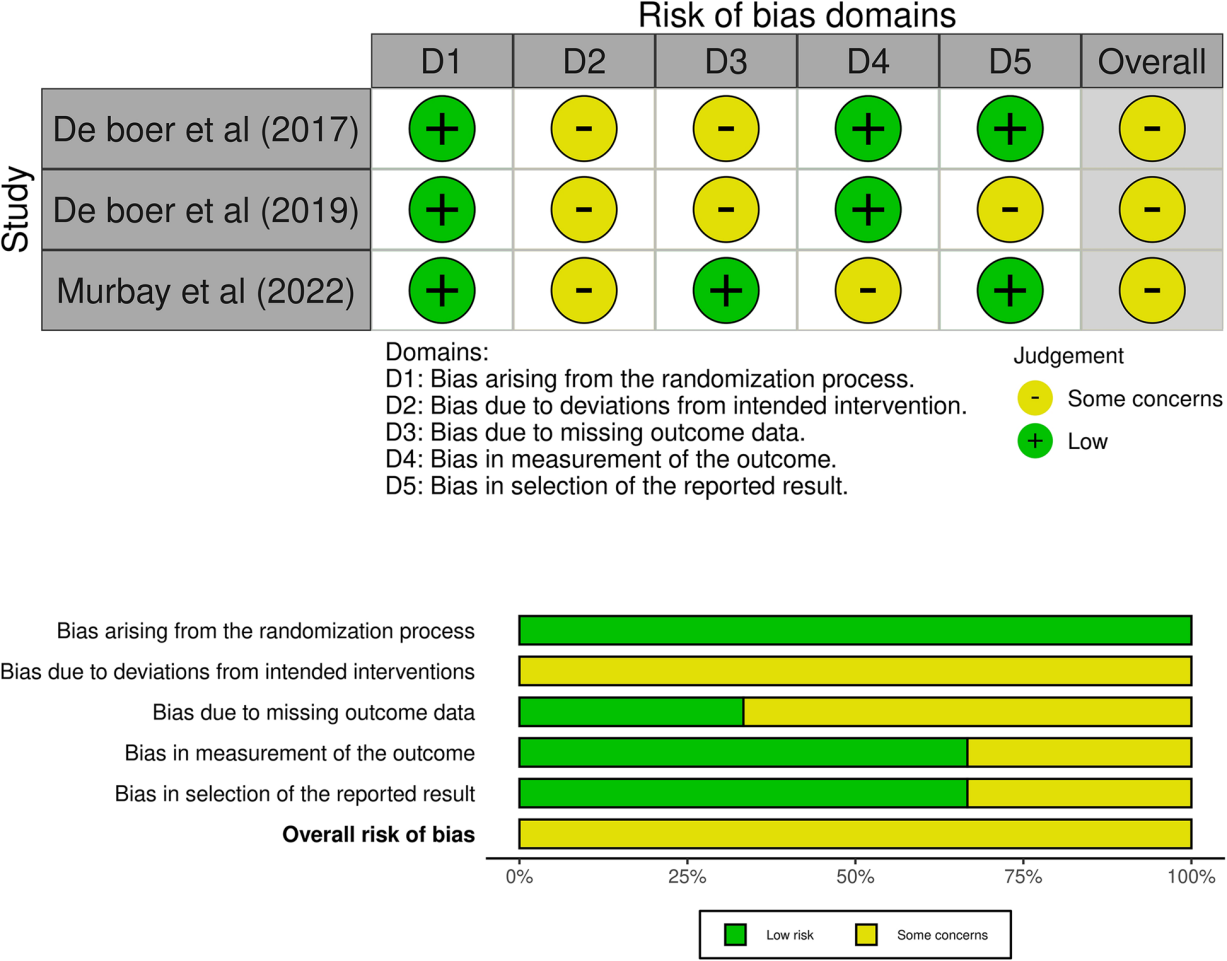
Summary of risk of bias assessment for randomised studies (RoB-2)

**Fig. 3:**
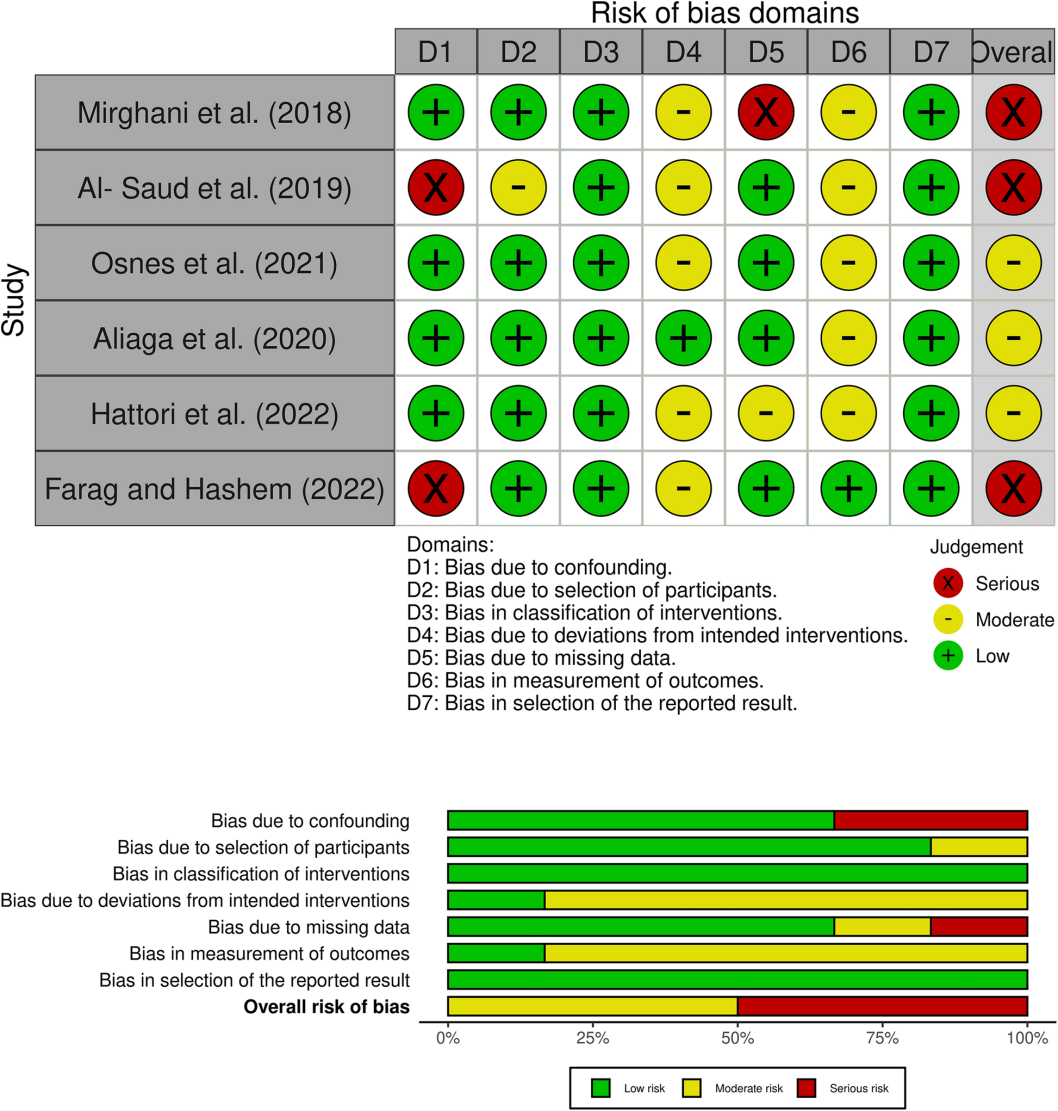
Summary of risk of bias assessment for non-randomised studies (ROBINS-I)

Most studies have considerable problems owing to unclear and insufficient reporting. Three out of nine studies showed a high risk of bias, while six studies, including three randomised trials, showed some concerns. Poorly reported items across the studies included sample size calculation and report of losses to follow up, raising apprehension about reliability and validity reflected in the higher risk of bias ratings. A summary of the risk of bias assessment is shown in Figs. 2 and 3 [34].

### Quality of evidence for outcomes in Summary of Findings table

Low-certainty evidence implies that Simodont training may have a favourable impact on dental students' psychomotor skill acquisition and development, enhancing motor abilities, manual dexterity, and clinical performance when compared to traditional training. The evidence was downgraded by two steps, due to bias and the fact that majority of the studies were non-randomised. The summary of findings is shown in Table 3.

**Table 3:** Summary of findings table

Quality assessment						Summary of findings		
**Outcome**	**Risk of bias**	**Inconsistency**	**Indirectness**	**Imprecision**	**Publication bias**	**Impact**	**No. of participants (Studies)**	**Certainty of evidence (GRADE)**
Improved Student performance and skill development	Serious^a^	Not Serious	Serious	Not serious	Not serious	Our confidence in the effect estimate is limited: the true effect may be substantially different from the estimate of the effect	826 (9)	Low ⊕⊕

^a^ Three showed high risk of bias; six studies were rated to have some concerns

### Characteristics of the selected studies

#### Study population and setting

The participant demographics and study settings varied, encompassing undergraduate dental students at different stages of their training and institutions located across different regions of the world. The studies were conducted on undergraduate dental students enrolled on the dentistry programme in their first [23, 24, 26, 28, 29], second [25, 27], third [24, 29, 31], fourth [24], fifth [24] and sixth [30] years of training. All the studies took place at various dental colleges and universities across the world with a majority being done in Europe (UK [24, 25, 28], Netherlands [23, 26]) and Asia (Hong Kong [27], Spain [29], Japan [30], and Saudi Arabia [31]).

#### Duration

The study duration varied from practically no pretraining to an exercise period of fifteen minutes to a longitudinal study where students who completed the first year were followed for 2 years and re-evaluated in the third year [24, 28, 29]. Another study followed up with students in their 2^nd^year to 2 years later when they performed on patients [25].

#### Pre-training

The duration and intensity of practice varied across studies, with some studies incorporating short practice sessions while others implemented longer and more extensive practice periods. Hattori et al*.* gave the participants free practice time of 10 min to become acquainted with the device, followed by crown preparation [30]. Farag and Hashem permitted the the students to practise for 20 min per day for four weeks [31]. De Boer et al*.* allowed participants to practice for three months with a standard amount of force feedback (FFB) to enhance their fine motor skills [26]. In another study students underwent four sessions of 45 min each before their test session [23].

#### Methodology used

Most studies used various versions of the Simdont such as the Moog Simodont dental trainer (Nieuw-Vennep, the Netherlands) [23, 26, 27, 29] with Simodont ‘courseware’ software (developed by the Academic Centre for Dentistry Amsterdam (ACTA), Amsterdam, Netherlands) [24, 25, 31] or Simodont (Nissin Dental Products Europe BV, Nieuw- Vennep, Netherlands) [30]. Osnes et al*.*did not mention the version of Simodont used [28].

### Outcome measure assessment

Seven out of the nine studies measured the efficacy of Simodont in restorative work either by operating on the standard caries removal protocol or the cutting of some form of geometric shape [23–27, 29, 31]. Real time feedback on performance was presented on a computer monitor which was assessed by experienced trainers [23–27, 29–31]. For a few studies the conventional simulator preparations were compared to those done using simodont [25, 27, 31]. Studies used various factors for evaluation such as target, error scores, drill time [24] or procedures done at different levels of force feedback [23, 26]. One study compared work done on the Simodont by experienced clinicians to that done by dental students [28].

Two studies measured the efficacy of Simodont in crown preparation [25, 30]. Their performance on patients was compared to their performance in VR and the conventional typodont 2 years prior [25]. Scores for preparations of the occlusal surface, margin design, surface smoothness, taper angle, total cut volume and overall impression of the products for both the conventional simulator and Simodont were compared [30].

### Effect of intervention

Nine studies were reviewed to evaluate the heterogeneity of results and to determine if Simodont is a valid tool for preclinical undergraduate education. Out of the nine studies, eight reported that Simodont is a valid tool for training dental students [23–29, 31], while Hattori et al*.*reported lower scores for students performing tooth preparation using the haptic simulator compared to the conventional method [30].

Studies found that the effect of force feedback was important in achieving high precision tasks in dentistry. Students practicing with the effect of force feedback outperformed those practicing without it [23]. Manual dexterity skill was found to be transferable from one level of force feedback to another if the students practiced for a sufficient amount of time [26]. On comparing work done by experienced and novice students the authors reported that Simodont showed sensitivity to performance differences between the two thus can be used for measuring dental performance and student education [24]. Studies comparing performance on conventional simulators versus haptic simulators showed that incorporating Simodont training would be a valuable adjunct in dental education [25, 27, 29, 31].

## Discussion

Haptic feedback devices have emerged as valuable tools in dental education, offering a three-dimensional virtual reality environment that replicates real dental settings. This controlled environment allows students to practice diverse dental procedures, and their effectiveness in preclinical training has been an ongoing subject of research and discussion [23–28, 31, 35, 36]. Through a comprehensive analysis of nine selected studies, this systematic review focused on assessing the impact of adopting Simodont in preclinical dental training, with a particular emphasis on the development of psychomotor skills, motor skills, manual dexterity, and clinical performance.

Studies reported that Simodont assisted in the acquisition and retention of fundamental psychomotor abilities needed for performing operative dentistry, particularly when combined with instructor feedback [25]. However, it's worth noting that not all studies were unanimous in their support for haptic simulators, with some reporting lower scores compared to conventional training for specific evaluation items. The differences in hand manipulation during preclinical procedures in the simulators may have contributed to the differences in students’ performance. Furthermore, individual differences in the depth perception ability and different retinal disparities may also have lead students to find depth perception difficult in the simulator [30]. The distinctive attributes of Simodont such as the generation of three-dimensional images through stereo viewers, have a discernible impact on the performance of operators and the perception of evaluators. Hence, it is imperative to design curriculum that takes into account such features offered by each simulator [30]. Overall, majority of studies reported the positive potential of Simodont as a valid tool for enhancing motor skills, manual dexterity, and clinical performance [23, 24, 26–28, 31].

The force feedback feature in haptic technology emerged as a critical aspect, enabling students to achieve the high precision levels necessary for manual dexterity tasks in dentistry [23]. Skills learned in virtual reality (VR) can be translated to real-world situations when students practise for long enough at one level of force feedback and then go on to the next [26]. The continuous evaluation of students using haptic simulators, along with sensory feedback during the preparation of enamel and dentine, enhanced hand–eye coordination and fine psychomotor control, thereby improving their psychomotor skills [31]. Simulation exercises were particularly valuable in assessing the students’ grasp of the concept of caries removal [28]. The results provide important implications for the use of Simodont in preclinical training of dental students. The sensitivity of Simodont in detecting performance differences between novice and experienced students suggests that it is a useful tool for measuring dental performance and student education [24].

Simodont's advantages could extend beyond the technical aspects, as it has the potential to reduce anxiety levels among dental students. The immediate feedback provided by haptic devices promotes self-assessment, allowing students to identify areas for improvement [37, 38]. Moreover, the ability to repeat procedures on haptic devices until acceptable skill levels are demonstrated without risking actual patients, can improve student confidence and competence and help in facilitating patient safety. Ethical decision-making training offered by Simodont enables students to navigate complex patient situations responsibly and ethically, enhancing overall patient care. This type of training provides unlimited reproducibility, objective evaluation of preparation by computer assessment, and cost reduction. It also narrows down the gap between preclinical and clinical skill levels [39]. Overall, Simodont proved efficient in training dental students in hand–eye coordination and spatial reasoning skills, improving preparation accuracy and shortening preparation time [40–44].

Multiple studies have reported that instantaneous feedback on student performance improved self-assessment, adaptation and eliminated subjectivity [40–42]. This finding corroborates earlier findings by *Vincent *et al*.* where haptic simulators could monitor and guide the progression of novices during cavity preparation [45]; though the role of teacher and verbal instructions cannot be ruled out [36]. VR simulators have become popular due to their ability to provide high-quality education, decrease inequality, and reduce waste [46]. However, patient oral environments of gingival tissues, saliva, tongue movements, and reflexes, such as gagging, cough, and head movement simulation, still need to be incorporated for better skill training and teaching emergency management [47].

It is recommended that students practice for a sufficient amount of time to ensure transferability of the skill in real-life situations. Urbankova et al*.* suggested that eight hours of computerized dental simulation training delivered early in the preclinical operative dentistry course is required to improve students' performance [48]. Data from virtual simulators can help stratify dental students and predict their clinical performance, providing an opportunity to tailor the learning process to meet individual diversity in students' expertise and allow students to work at their own pace, thus helping them reach optimal performance [36].

A major strength of this review is the comprehensive search strategy, which aimed to identify all relevant studies on virtual Objective Structured Clinical Examination (OSCE) in dental education. The use of two independent reviewers in study selection, data extraction, and quality assessment also enhances the reliability of the findings. However, the review also has several limitations that need to be considered. The number of studies included in the review is relatively small, and the sample sizes of the individual studies are generally low, which may affect the generalizability of the findings. Additionally, the study designs and assessment methods used in the included studies were not standardized, which limits the ability to draw definitive conclusions. The studies included in the review also had a moderate to serious risk of bias, which may affect the validity of the findings. Furthermore, the review only focused on Simodont in dental education, limiting the generalizability of the findings to other haptic training devices. Given the limited availability of data, these results warrant cautious interpretation. A majority of studies were conducted in first world countries with no studies conducted in lower and middle-income countries (LMICs) where where infrastructural resources may be relatively scarce. Hence, larger datasets are required to validate and replicate these findings, which could potentially contribute to the assessment, design, and targeting of haptic interventions.

Overall, Simodont has the potential to be an effective and accepted adjunctive training method in dental education, but further research is needed to determine its full impact. Overall, our review addresses the need for dental educators to adopt new and innovative methods of teaching preclinical skills to dental students, and provides valuable insights into the potential benefits of haptic feedback devices in this regard. The findings of this review may inform educators and policymakers about the potential benefits of Simodont haptic feedback devices as a teaching tool for preclinical dental training. The incorporation of Simodont can bridge the gap between preclinical simulation and real-world clinical practice, enhancing the preparation of dental students for patient care. Given the potential benefits and positive outcomes observed in the selected studies, further research, and collaboration between dental educators and Simodont developers are essential to maximize the impact of this technology on dental education and, ultimately, improve patient outcomes [49–57].

## Conclusions

This systematic review evaluated the effectiveness of Simodont in the preclinical training of dental students. Based on the limited evidence available, there is low-certainty evidence that Simodont is effective in improving the motor skills, manual dexterity, and clinical performance of dental students. The effect of force feedback feature is important to acquire manual skills and if practised for long enough these skills can be transferred to reality. While acknowledging the limitations in reporting and study designs, the majority of the reviewed studies highlight the value of Simodont in preclinical dental education. However, well-planned high-quality studies with larger sample sizes are required for further evaluation of the assessment methods.

## Data Availability

Prof. Shankargouda Patil will have access to the data that were the basis for this article, and can be reached out for data in case is needed for review.
